# A Functional Variant in *ERAP1* Predisposes to Multiple Sclerosis

**DOI:** 10.1371/journal.pone.0029931

**Published:** 2012-01-12

**Authors:** Franca Rosa Guerini, Rachele Cagliani, Diego Forni, Cristina Agliardi, Domenico Caputo, Andrea Cassinotti, Daniela Galimberti, Chiara Fenoglio, Mara Biasin, Rosanna Asselta, Elio Scarpini, Giacomo P. Comi, Nereo Bresolin, Mario Clerici, Manuela Sironi

**Affiliations:** 1 Don C. Gnocchi Foundation ONLUS, Milano, Italy; 2 Bioinformatic Laboratory, Scientific Institute IRCCS E. Medea, Bosisio Parini, Italy; 3 Department of Clinical Sciences, Chair of Gastroenterology, Luigi Sacco University Hospital, Milano, Italy; 4 Dino Ferrari Centre, Department of Neurological Sciences, University of Milano, Fondazione Ca' Granda IRCCS Ospedale Maggiore Policlinico, Milano, Italy; 5 DISP LITA Vialba, University of Milano, Milano, Italy; 6 Department of Biology and Genetic for Medical Science, University of Milan, Milan, Italy; 7 Department of Biomedical Sciences and Technologies LITA Segrate, University of Milan, Milan, Italy; Institute Biomedical Research August Pi Sunyer (IDIBAPS) - Hospital Clinic of Barcelona, Spain

## Abstract

The *ERAP1* gene encodes an aminopeptidase involved in antigen processing. A functional polymorphism in the gene (rs30187, Arg528Lys) associates with susceptibility to ankylosying spondylitis (AS), whereas a SNP in the interacting *ERAP2* gene increases susceptibility to another inflammatory autoimmune disorder, Crohn's disease (CD). We analysed rs30187 in 572 Italian patients with CD and in 517 subjects suffering from multiple sclerosis (MS); for each cohort, an independent sex- and age-matched control group was genotyped. The frequency of the 528Arg allele was significantly higher in both disease cohorts compared to the respective control population (for CD, OR = 1.20 95%CI: 1.01–1.43, p = 0.036; for RRMS, OR = 1.26; 95%CI: 1.04–1.51, p = 0.01). Meta-analysis with the Wellcome Trust Cases Control Consortium GWAS data confirmed the association with MS (p_meta_ = 0.005), but not with CD. In AS, the rs30187 variant has a predisposing effect only in an *HLA-B27* allelic background. It remains to be evaluated whether interaction between *ERAP1* and distinct *HLA* class I alleles also affects the predisposition to MS, and explains the failure to provide definitive evidence for a role of rs30187 in CD. Results herein support the emerging concept that a subset of master-regulatory genes underlay the pathogenesis of autoimmunity.

## Introduction

Antigen processing and presentation by MHC class I molecules is essential for assuring immune surveillance and for establishing immunodominance. The process initiates with the transport of proteasome-generated antigenic peptides to the endoplasmic reticulum (ER), where they are customized to optimal size for MHC class I loading by resident enzymes. In humans, two ER-aminopeptidases, encoded by *ERAP1* and *ERAP2*, trim imported peptides at their N-terminus and contribute to the shaping of the antigenic repertoire presented by class I MHC molecules [1]. Studies in humans and mice have shown that, depending on peptide length and sequence composition, ERAP1 has the ability to both destroy and create peptide cargos for MHC class I [2]. Therefore, in mice lacking the enzyme the presentation of some peptides is dramatically reduced, whereas other peptides are much more abundant than what is observed in wild-type animals [3]. This applies to both proteolytic fragments of pathogen-derived proteins and to endogenous peptides. As a consequence, immunodominance is disrupted in *Erap1^−/−^* mice and these animals display a distinct repertoire of antigenic peptides [3].

Because ERAP1 also contributes to shedding the membrane-bound receptor for inflammatory cytokines including IL1R2, TNFR1, and IL6R [4], ERAP1 is likely to play a pivotal role in protection from infectious diseases, in maintaining immunotolerance, and in controlling inflammation. A single nucleotide polymorphism (SNP) in *ERAP1* (rs30187), which changes a highly conserved residue (Arg528Lys), is maintained at intermediate frequency in human populations by natural selection [5] and affects the enzyme catalytic activity [6]. This SNP has been associated with susceptibility to ankylosying spondylitis (AS) [7], and variants in linkage disequilibrium (LD) with it increase predisposition to psoriasis [8]. This observation is in line with an emerging concept whereby a portion of susceptibility alleles is shared among two or more autoimmune conditions (reviewed in [9]), suggesting that a subset of master-regulatory genes underlay the pathogenesis of autoimmunity, although the clinical outcomes and end-organ targets differ across diseases. For example, variants in *IL23R* have been associated with psoriasis, AS, and Crohn's Disease (CD). Additional shared variants between CD and AS have recently been described [10], [11], and provide genetic evidence to the clinical observation that the two diseases have frequent co-occurrence and co-symptomatology [12].

Thus, we wished to verify whether the *ERAP1* susceptibility allele for AS also predisposes to CD and MS, this latter also showing some degree of co-morbidity with Crohn's disease in affected individuals and their family members [13]–[15].

## Results and Discussion

Multiple SNPs in *ERAP1* have been associated with AS, but the strongest signal is accounted for by rs31087 (Arg528Lys) [7]. As mentioned above, the variant was recently shown to be functional by affecting both peptide trimming and antigen presentation [6], [7]. Thus, we focused on this SNP and set out to verify whether it may affect the predisposition to CD and MS. To this aim, rs30187 was genotyped in 572 patients with CD and in 517 subjects suffering from relapsing-remitting MS (RRMS); two independent cohorts of sex- and age-matched controls were also analysed. All individuals were Italian of European ancestry and the SNP complied to Hardy-Weinberg equilibrium in the case and control cohorts.

The genotype and allele distributions of rs30187 are shown in Table 1 for both CD and RRMS patients compared to two independent healthy control (HC) cohorts. Statistically significant associations of rs30187 genotype and allele distributions were observed both in CD and in RRMS.

**Table 1:** Association study and meta-analysis for rs30187 in RRMS and CD.

Disease	Genotype Counts(GG/AG/AA)		*P_geno_*	Allele Counts(A/G)		*P_allelic_* a	OR (95% CI)	Meta-analysis			
	Cases	Controls		Cases	Controls			Het. *P*-value^b^	*I^2^* c	*P_meta_* d	OR_meta_
RRMS	182/254/81	209/233/58	0.043	416/618	349/651	0.014	1.26 (1.04–1.51)	0.29	8.7	0.005	1.16
CD	211/275/86	247/273/68	0.094	447/697	409/767	0.036	1.20 (1.01–1.43)	0.02	81.1	0.58	1.06

a P value from Pearson's Chi-squared test with Yates' continuity correction.

b P value from Cochran Q heterogeneity test.

c Heterogeneity index.

d Random-effects meta-analysis p value.

In particular, the AA genotype was more frequent both in CD patients (15.0%, CD vs. 11.5%, HC) and MS subjects (15.7%, MS vs. 11.6%, HC) compared to their respective control samples, and a statistically significant association of the rs30187 A allele was observed both in CD (odds ratio, OR: 1.20; 95% confidence interval, CI: 1.01–1.43) and in MS patients (OR: 1.26; 95% CI: 1.04–1.51) (Tab. 1). Thus, the minor A allele of rs30187 (528Arg), previously associated with AS, also confers susceptibility to CD and MS in these Italian cohorts. In order to perform a meta-analysis, we exploited genome-wide association study (GWAS) data for MS and CD generated by the Wellcome Trust Cases Control Consortium (WTCCC1 project data). As estimation of effect heterogeneity is inaccurate when few studies are included in the meta-analysis, we applied a random-effects model [16]. rs30187 was not genotyped in the CD GWAS; a search for linked SNPs identified rs27710, which has been genotyped by the WTCCC1 and is in full LD with rs30187 in the Italian population (r^2^ from the 1000 Genomes Project data for TSI = 1), making imputation straightforward. As for MS, rs30187 was available in the GWAS study. Random-effect meta-analysis with these data supported the association between rs31087 and MS susceptibility (p_meta_ = 0.005) (Tab. 1). Conversely, high between-study heterogeneity was observed for CD, resulting in failure to confirm the association we observed in the Italian sample (Tab. 1). It is worth mentioning that the p value obtained for MS after meta-analysis does not reach the GWAS statistical threshold, suggesting the need to further replicate this association in independent studies.

AS and CD are known to have a close clinical relationship: about 10% of AS patients also suffer from inflammatory bowel disease, and most AS cases display evidence of chronic intestinal inflammation [12]. Arthropathyes are common among CD patients as well [17]. Consistently, risk alleles that predispose to both conditions have been recently identified [10], [11]. A non-synonymous variant in *ERAP2* (rs2549794), which acts in concert with ERAP1 in the ER, has been associated with the risk of CD in a GWAS [10]. Although the two aminopeptidase genes are located in a cluster on chromosome 5, rs30187 and rs2549794 segregate independently, as the two SNPs display extremely limited LD both in Italians [5] and in HapMap populations of European ancestry (*r^2^* = 0.18, http://hapmap.ncbi.nlm.nih.gov/). These observations make *ERAP1* a good candidate as a susceptibility gene for CD. Our analysis in the Italian population supported the role of the A allele of rs31078, which predisposes to AS, in susceptibility to Crohn's disease; nonetheless, this finding was not supported when data from a second study were used for meta-analysis. One possibility is that the *ERAP1* variant genetically interacts with specific *HLA* class I alleles. Indeed, in the case of AS, rs30187 was shown to display a strong genetic interaction with *HLA-B27*, which is extremely common in spondylitis patients [7]. This observation suggests that the co-occurrence of the 528Arg allele at *ERAP1* and *HLA-B27* results in the presentation of antigenic species that prompt disease pathogenesis. Similar observations have been reported for psoriasis, as variants in *ERAP1* have a predisposing effect only when combined with specific *HLA-C* allelic backgrounds. No specific *MHC* allele/haplotype has been reported in CD, although several significant associations have been described for SNP alleles within the *MHC* [18]. Therefore, the role of *ERAP1* alleles in the pathogenesis of CD remains to be evaluated, as well as the presence of possible epistatic effects of *HLA* alleles.

Recent findings have indicated that a portion of susceptibility alleles for autoimmune disease is shared among two or more conditions (reviewed in [9]). Our data indicate that the AS susceptibility allele in *ERAP1* also confers increased risk to develop MS, and imply a role for antigen presentation and class I MHC molecules in the pathogenesis of MS. The strongest genetic risk factor for MS is the HLA DRB1*1501-DQB1*0602 haplotype (also known as DR15 haplotype) in the *HLA*-class II region. In Italians, as well as in other European populations [19]–[21], DR15 confers an OR of about 3. Yet, in recent years, it has been suggested that the HLA-class I region does indeed exert an additional influence on the risk of MS, analogous to that reported for other autoimmune diseases [22]–[24], and with an effect independent from *HLA-DRB1* [25]. Again, further analyses will be required to verify whether the *ERAP1* Arg528Lys variant interacts with specific *HLA* class I alleles to modulate predisposition to MS.

As mentioned above, in addition to its role as an ER-aminopeptidase, ERAP1 also functions as a cleavage enzyme for IL1R2, TNFR1 (also known as TNFRSF1A), and IL6R. *TNFRSF1A* is a susceptibility locus for MS and CD [26], [27], and variants in *IL1R2* have been associated with AS and ulcerative colitis [7], [28], while IL6 is a central mediator of inflammation. Thus, the associations we detected between *ERAP1* and MS might relate to the role of the enzyme as a receptor sheddase, although it is presently unknown whether the Arg528Lys also affects this cleavage activity.

In summary, we report that a functional *ERAP1* allele previously associated to AS confers susceptibility to MS in Italian populations, whereas its role in predisposing to CD remains to be evaluated. Thus, results herein add further support to the shared genetic architecture of autoimmune diseases.

## Materials and Methods

For the MS case/control association study, a total of 1017 individuals were enrolled: 517 patients (343 females and 174 males) suffering from RRMS and 500 age- and sex-matched healthy controls (325 females and 175 males) were recruited at the MS Centre of Don Gnocchi Foundation in Milan and at Department of Neurological Sciences, University of Milan. All subjects gave informed consent according to protocols approved by the local Ethic Committees. All patients and controls were Italians of European origin. Patients underwent a standard battery of examinations, including medical history, physical and neurological examination, screening laboratory test, and brain Magnetic Resonance Imaging (MRI). Patients with RRMS fulfilled the McDonald's criteria [29]. Median age was 42.1+11.9 and 43.12+18.22 years for RRMS and controls, respectively.

For the CD case/control cohorts, 1160 individuals: 572 suffering from CD (301 males, 271 females) and 588 age- and sex-matched healthy individuals (305 males, 283 females) were recruited by the IBD Unit of the Luigi Sacco Hospital in Milano, a third-level centre for the management of IBD patients. The diagnosis of CD was based on international published criteria, according to clinical, endoscopic, histological and/or radiological data [30]. A detailed clinical history, as well as laboratory and instrumental diagnostic data, were collected. Also in this case, all patients and controls were Italians of Caucasian ethnicity.

Genotyping of rs30187 was performed by a TaqMan probe assay (TaqMan SNP genotyping assay, Applied Biosystems, Foster City, CA, USA) using the allelic discrimination real-time PCR method.

Genotype data for rs30187 and rs27710 from the WTCCC1 studies has been retrieved from the European Genome-phenome Archive (EGA, http://www.ebi.ac.uk/ega/) which is hosted by the EBI, under accessions EGAS00000000006 (CD) and EGAS00000000022 (MS). For meta-analysis, we applied a random-effects model as implemented in PLINK [31].
